# A Freak of Nature

**Published:** 1888-03

**Authors:** 


### A FREAK OF NATURE.

Fred Beck, a saloon keeper on Pearl and Lawrence streets, is the possessor of three most remarkable specimens of animal malformation. Some time ago Beck came into possession of a little canine having but two legs. The places where the fore legs should be are marked by little stumps, perhaps an inch long. The little animal was the source of much comment on account of its singular malformation. A cross between a black and tan and water spaniel, the strangely formed little thing is possessed of more than ordinary intelligence. About the place "Jennie" has been the cause of much curiosity. The absence of her fore feet do not interfere materially with her locomotion, however. Nature having deprived her of fore pedal extremities, has endowed her with the power to use two limbs with remarkable activity, thus compensating her for the loss of the other two. When spoken to, the little thing will sit on end like a rabbit, and jumps and waddles about something after the fashion of that animal. Sometime ago Jennie gave birth to a litter of pups, all of which were perfect in form. This was not at all remarkable, however. Several days ago she again became a mother. Three pups were born. One of them is perfect, but the other two are as remarkable as Jennie herself, so far as the absence of legs is concerned. One has three legs and the other but two, like its mother. The remarkable freak of nature did not become known to Beck for a couple of days, as by chance every time the pups were looked at the one with four legs was picked out. Finally, however, the real state of affairs was discovered. The pups are all alive and well The mother seems to realize that her little family is the subject of much curiosity, and is as gentle and kind as it is possible for her to be when visited by strangers desirous of looking at her and her remarkable little ones.—*Cincinnati Enquirer.*

